# The discomfort of an educator’s critical conscience: the case of problem-based learning and other global industries in medical education

**DOI:** 10.1007/s40037-016-0325-x

**Published:** 2017-01-03

**Authors:** Janneke M. Frambach, Maria Athina (Tina) Martimianakis

**Affiliations:** 1 School of Health Professions Education, Maastricht University, Maastricht, The Netherlands; 2 Department of Paediatrics and The Wilson Centre, University of Toronto, Toronto, Canada

Halman, Baker and Ng’s article [1] on critical consciousness captured our attention as scholars interested in engaging in educational practices that are dynamic and stay responsive to local needs. We expound on ways in which it has made us think about our practice, specifically in the face of globalization in health professions education. We argue that our practice as health professions educators is inextricably linked to a global industry of health professions education, which comes with a potentially discomforting message.

## Critical consciousness: a pedagogy for students and teachers

Educational platforms such as competency-based medical education (CBME) run ‘the risk of reducing learning to a series of measurable skills and behaviours’, Halman et al. caution in this issue [1]. The authors advocate for a critical approach to medical education that moves beyond ‘the rigid categories of knowledge, skills and attitudes’, which they claim fail to represent the diversity of ways of knowing, thinking, being and acting that we need from our health professionals today. Awareness of a patient’s personal history and community; of social determinants of health; of power structures and how they impact health and healthcare systems; and, most importantly, social action and transformation resulting from this awareness are things the authors note cannot simply be translated into neatly described, measurable competencies.

A risk of such calls for critical and social awareness is a failure to provide micro-level guidance for teachers and course designers: ‘Sounds great, but how do we do this?’ Halman et al. anticipated this question and list practices common to critical pedagogy, such as promoting authentic dialogue and challenging hierarchies [1]. Still, educators might be left wondering how to incorporate these in their teaching practice. The authors indeed emphasize their paper is an early step in incorporating critical consciousness as a pedagogy in health professions education, and that more investigation is needed of how to position this pedagogy alongside existing educational approaches and theories. In fact, they warn educators not to adopt these practices in existing curricula without analyzing if and how the theoretical underpinnings of critical pedagogy and existing approaches are reconcilable.

On the surface, several prominent educational approaches currently used in health professions education seem to fit well with the shift away from the banking model of education (where the teacher simply deposits predetermined important education into the learner), and the adoption of critical consciousness promoted by Halman et al. Take problem-based learning (PBL). It radically transformed the status quo of medical education in Canada in the late 1960s and of multiple other contexts thereafter. Its key features include student-directed dialogue centred on (authentic) cases and a diminished hierarchical distance between students and tutors. Potentially, PBL may provide a nurturing space for the five key themes Halman et al. determined as linked to critical consciousness: appreciating context, illuminating power structures, moving beyond the procedural, enacting reflection, and promoting equity and social justice [1].

However, a deeper investigation of PBL’s roots and theoretical underpinnings, dominantly based in cognitive psychology, [2] makes its compatibility with critical consciousness more doubtful. PBL’s educational objective was never to challenge explicitly existing structures or promote equity, but to serve students’ acquisition of knowledge and skills [3]. The bulk of research on PBL investigates if and how it ‘works’ – i. e. leads to higher levels of knowledge and skills compared with alternative approaches – and how it can be optimized in different circumstances [4–6]. Nowadays, because of its pervasiveness, PBL is rarely perceived as transformative and has, in fact, been noted for its potential to perpetuate existing power structures and limit students’ critical perspectives [7, 8]. Reconciling existing educational approaches such as PBL with critical pedagogy would require that both teachers and learners are empowered to challenge not only what is being learned, but why this learning is expected of them and how it is being delivered.

In other words, this would ask from us a willingness to challenge the assumptions underpinning our work as educators, or as Halman et al. put it: ‘a willingness to challenge one’s own position of power and privilege’ [1]. If we intend to ask this from our students as future health professionals, we obviously should be prepared to face this challenge ourselves too. What if our students disagree with the premise of our knowledge? What if our colleagues in another context fail to acknowledge our starting or endpoints as valid? Critical consciousness is not a concept relevant only to health and health professionals, but to education and educators just as much. Introducing critical approaches to health professions education cannot ignore the power structures of our education systems, specifically the social and political determinants of medical education. Are we critically conscious about our educational approaches? And are we ready to challenge them if our own position, and those of our co-workers, depends on them?

## The social and political determinants of global medical education

To proceed with the case of PBL: the first author’s affiliation, Maastricht University in the Netherlands, is generally considered a PBL ‘guru’ in the global field of medical education, and has been identified as the ‘single most prolific producer of empirical research on PBL in the method’s 50-year history’ [2]. The global PBL industry is big; the most heavily cited research in medical education is on PBL, [9] and a plethora of trainings and consultancies on PBL are offered worldwide. While we use this as an example, the global industry of PBL is not an isolated phenomenon. Learning and assessment tools such as OSCE, EPAs and simulation, competency instruments such as CanMEDS, accreditation frameworks such as ACGME-International and many other tools are key commodities in the medical education marketplace [10]. In the face of increasing globalization of health professions education, so called standardized and validated educational tools provide efficiency in the transport of knowledge across contexts, but in the process can inadvertently reproduce dominant educational power structures and obfuscate important local and context specific approaches to health professions education.

Continuing with our case example, PBL has been promoted as an educational approach that delivers self-directed, lifelong learners equipped with a holistic set of professional and social skills ready to face twenty-first century healthcare [11]. Publications on global standards and guidelines in medical education list these skills as essential attributes of health professionals globally, [12–14] which is further reinforced by a rich representation of research on such aspects in high impact medical education journals. As such, and notwithstanding intense debates in the past decades on PBL’s effectiveness, the discourse in medical education worldwide is largely favourable to the PBL movement – as well as to other key medical education commodities mentioned above, such as CBME. This global discourse catalyzes motivation, drives national accreditation standards, and provides justification and funding opportunities for PBL implementation, as well as avenues for institutions to profile themselves against national and international competitors [10]. Social and political forces such as these have contributed to a worldwide spread of PBL and have made it difficult for some institutions to resist and/or to argue for context specific pedagogies, better suited to their circumstances [10].

As a result, PBL currently appears in places where other approaches might have been more fruitful, and where specifics of the context might have been overlooked or ignored. While praised for its effectiveness in different settings around the world, PBL has often been critiqued in different settings around the world as well: for its costs and demands on human resources, the efforts involved in implementing and sustaining the approach, its relation to the cultural context, its role in the transmission of distinctive educational values and interests, and its potential to work against, rather than support local educational needs and priorities [10]. Similar critiques and reflections exist of other commodities in the global medical education industry, notably from the perspective of post-colonial scholars [15–17]. The broad adoption of PBL worldwide has further enabled institutions who have historically been in a position to build capacity and experience in PBL and share their knowledge and research worldwide, such as Maastricht University, to continue being looked at for expertise in education more generally, possibly at the expense of others who did not have such a headstart or who have a different educational message that is less aligned with mainstream global discourse. Established institutions benefit from their reputation and offer consultancies to newcomers, from which the former’s reputation and budget continuously grow and the cycle continues. The big players moreover are in a position to influence the global discourse more than others. In the case of PBL, for example, it is important for key players whose identity and training are interconnected with a certain way of understanding and promoting the concept, that we continue to talk about PBL as ‘one thing’, while practice – and theory [5, 18] – shows this type of consensus is difficult to find and may run against the needs and interests of smaller players.

Being critically conscious about our educational approaches, then, moves beyond evaluating an approach’s ability to train critically conscious health professionals. It also involves thinking about the historical, social and political determinants that have generated our current training models. Where does this training model come from? How did I come to know it? Who benefits or loses, within and beyond my institution, when I use or promote this model? Questions such as these that serve to surface embedded assumptions, are fundamental starting points for critically conscious educators – but asking questions is not enough. Halman et al. emphasize that when it comes to critical consciousness ‘reflection without social action is insufficient’ [1]. A key objective of a critical approach is transformation of the status quo to a more equitable state. Philosophically many might be aligned with this ideal, but without practical guidance for educators, who may experience an ‘absence of perceived or actual agency’ [1] towards these macro-level issues, reflexivity will not lead to praxis. Moreover, action on these issues is scary, as it will often affect our own positions within our organizations and fields, particularly if our critique challenges practices that are perceived to be scholarly and valid. We continue our self-reflection by considering how we may attach a praxis to our critique. Admittedly, this part is more difficult.

## Moving from reflection to praxis

The first author is involved in the global industry of PBL as both a researcher and a trainer of PBL, as are many of her colleagues. How critical can and does she want to be about promoting PBL as an educational approach? Which actions can and does she want to take, knowing the role PBL plays in hers and others’ academic and professional careers, her employer’s global reputation, and revenues of an entire industry? She might design her PBL trainings differently; publish critical research on PBL and its globalization; open up space to discuss alternative approaches with PBL customers; consider referring them to other institutions; collaborate with smaller players to create joint training offers that reflect contextual diversity; reverse the flow of training and consultancy fees by participating in trainings offered by less well-known institutions; work within her institution to engage PBL faculty and students in critical reflection; and advocate for these issues among powerful educational leaders inside and outside her institution.

The second author is an education researcher working in a clinical department. Often she is faced with the difficult task of pointing to ways in which the day-to-day educational practices of her colleagues, and her own, inadvertently create inequities or marginalize perspectives. She might ask pointed questions during meetings; refuse to engage in an activity she sees as problematic; challenge her colleagues to disrupt routine practices and create forums for discussing alternative ways of thinking or acting. However, it’s one thing to speak about inequities in the abstract, and quite another to draw attention to local activities one finds problematic, while maintaining a productive role in the organization that pays your salary. The potential anxiety captured within such actions, ‘may paradoxically trigger emotionally distancing reactions and become a barrier to engagement’ [1]. Meaning, when it really comes to our own position and privilege, we often prefer to stay quiet and go with the flow. From our own experience, we want to acknowledge that reform from within is effortful and requires sacrifices. For example, there are many instances in the second author’s day that she engages in educational activities that contribute to the ‘selling’ and/or transmission of Canadian knowledge products around the world. While she may not always agree with the premise of this work, she engages in it with the goal and the hope of having future opportunities to contribute to reform from within.

It is obvious that critical consciousness will not come to us easily. On top of the already complex issues of embedding critical pedagogy within current curricula and providing course-level guidance, it involves first and foremost willingness from our part to ask painful questions, confront discomforting answers and take disruptive actions. Personally, we would love to proclaim that we are critically conscious educators, but the truth is that we have not measured up in all situations, as this would personally jeopardize our capacity to stay active as educators in our respective fields. We cannot ask our students to engage in critical consciousness alone, who, as also Halman et al. [1] note, are often in even more precarious positions than we are. They need us to be their role models. Critical consciousness, by definition, is a dangerous, uncomfortable educational practice that only works if we never expect it to become a safe practice. We still have a long way to go before we can declare that our field can engage in productive negotiations around what constitutes the ‘right’ way to educate health professionals. But we note that the time and space required for such negotiations is not outside our reach, and the steps outlined by Halman et al. are an important starting point for improving the quality of our educational programs.
